# Quasi‐Copper‐Mers Enable High‐Performance Catalysis for CO_2_ Reduction

**DOI:** 10.1002/advs.202303297

**Published:** 2023-08-08

**Authors:** Jing Yang, Ximeng Liu, Zhao Li, Shibo Xi, Jianguo Sun, Hao Yuan, Weihao Liu, Tuo Wang, Yulin Gao, Haimei Wang, Junjie Wang, Jun Song Chen, Rui Wu, Yong‐Wei Zhang, John Wang

**Affiliations:** ^1^ Institute of High Performance Computing (IHPC) Agency for Science, Technology and Research (A*STAR) 1 Fusionopolis Way, #16‐16 Connexis Singapore 138632 Singapore; ^2^ Department of Materials Science and Engineering National University of Singapore Singapore 119077 Singapore; ^3^ School of Materials and Energy University of Electronic Science and Technology of China Chengdu 610054 China; ^4^ Institute of Sustainability for Chemicals Energy and Environment (ISCE2) Agency for Science, Technology and Research (A*STAR) 1 Pesek Road, Jurong Island Singapore 627833 Singapore; ^5^ National University of Singapore (NUS) Research Institute (Chongqing) Chongqing Liang Jiang New Area Chongqing 401120 China

**Keywords:** CO_2_ reduction, copper atom, quasi trimer

## Abstract

As the atmospheric carbon dioxide (CO_2_) level keeps hitting the new record, humanity is facing an ever‐daunting challenge to efficiently mitigate CO_2_ from the atmosphere. Though electrochemical CO_2_ reduction presents a promising pathway to convert CO_2_ to valuable fuels and chemicals, the general lack of suitable electrocatalysts with high activity and selectivity severely constrains this approach. Herein, a novel class of electrocatalysts is investigated, the quasi‐copper‐mers, in which the CuN_4_ rather than Cu atom itself serve as the basic building block. The respective quasi‐copper‐monomers, ‐dimers, and ‐trimers hosted in a graphene‐like substrate are first synthesized and then performed both experimental characterization and density functional theory (DFT) calculations to examine their atomic structures, evaluate their electrocatalytical performance and understand their underlying mechanisms. The experimental results show that the quasi‐copper‐trimers not only outperform the quasi‐copper‐dimer and quasi‐copper‐monomer when catalyzing CO_2_ to CO, it also shows a superior selectivity against the competing hydrogen evolution reaction (HER). The DFT calculations not only support the experimental observations, but also reveal the volcano curve and the physical origin for the qausi‐copper‐trimer superiority. The present work thus presents a new strategy in the design of high‐performance electrocatalysts with high activity and selectivity.

## Graphical abstract

A combination of theoretical and experimental studies is carried out and discovers the quasi‐copper‐trimer has the highest activity and selectivity to catalyze CO_2_ into CO as compared to copper single‐atom and quasi‐copper‐dimer. It shows the combination of three Cu─N_4_ units, even without the direct bonding between Cu atoms, can still significantly enhance the CO_2_RR catalytical ability.

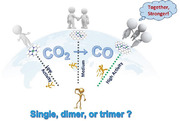

## 1 Introduction

Over the past two centuries, the global atmospheric average carbon dioxide (CO_2_) concentration has been steadily rising, and it has reached an unprecedented level of 420 ppm in April 2022. This has caused the climate change and even‐worsening environment, which are among the most threatening challenges faced by humanity in the 21st century.^[^ ^1^ ^]^ Developing a cost‐effective catalytic process for electrochemical CO_2_ reduction reaction (CO_2_RR) has been considered as a feasible solution to overcome this big challenge. Cu and Cu‐based materials stand out as the most promising electrochemical catalysts for CO_2_RR, because of their unique capability in producing a broad range of hydrocarbon fuels and chemicals at significant rates.^[^ ^2^ ^]^ Cu was among the first electrocatalysts reported that can catalyze CO_2_ to CH_4_ and C_2_H_4_.^[^ ^3^ ^]^ However, unmodified Cu exhibits rather poor selectivity and activity for formate production. Both entity size and morphology were found to play significant roles. For example, Cu nanocubes can substantially enhance the catalytic activity and selectivity for CO_2_ reduction, compared to Cu nanospheres of similar particle sizes, and remarkably they can reach a Faradaic efficiency of 60% and a partial current density of 144 mA cm^−2^ toward C_2_H_4_ production.^[^ ^4^ ^]^ Cu nanoparticles of sizes below 5 nm have shown a dramatic increase in the overall catalytic activity and the selectivity for certain hydrocarbons (methane and ethylene).^[^ ^5^ ^]^ Owing to the unique electronic structure and high atom utilization, single‐atom catalysts (SACs) have recently demonstrated unprecedented activity and selectivity for CO_2_RR, which holds great promise in reducing carbon emission and storing renewable energy.

The pioneer work on electrochemical CO_2_ reduction by SACs can be traced back to 1974 when Meshitsuka et al. reported that Co and Ni phthalocyanines on graphene substrate were active in CO_2_ electroreduction.^[^ ^6^ ^]^ Since then, metal–organic complexes, normally featured as M─N_4_ sites, have been widely investigated as a catalyst for electrocatalytic CO_2_ reduction with enhanced activity and selectivity.^[^ ^7^ ^]^ The M─N_4_ CO_2_RR catalysts were first reported in 2015, by Strasser et al. who demonstrated that Ni and/or Mn SACs catalysts were active and highly CO‐selective in CO_2_RR to CO/H_2_ mixtures, outperforming a low‐area polycrystalline gold benchmark.^[^ ^7b^ ^]^ Following on, this research field has been expanded rapidly, and several new single metal sites (such as Sn,^[^ ^8^ ^]^ Sb,^[^ ^9^ ^]^ Bi,^[^ ^10^ ^]^ Mo,^[^ ^11^ ^]^ Cu^[^ ^12^ ^]^ et al.) have been identified and different reduction products have been achieved, for example including CO,^[^ ^8^, ^10^ ^]^ formate,^[^ ^11^ ^]^ methanol,^[^ ^12^ ^]^ ethanol^[^ ^13^ ^]^ et al. These atomically dispersed metal sites usually act as the main active centers in the reported electrocatalysts; the coordinated atoms (mostly C and N) around the metal sites, on the other hand, are believed to facilitate the CO_2_ activation or the dissociation of the intermediates.

Although SACs possess impressive CO_2_RR‐catalyzing ability and selectivity, their further modifications are needed in order to achieve an even higher CO_2_RR performance toward practical applications in terms of activity, selectivity, energy efficiency, as well as long‐term stability. Motivated by the effectiveness of the paired or quasi‐paired metal atoms that were found to significantly enhance the electrocatalytical performance,^[^ ^14^ ^]^ herein we extended the Cu SAC to the quasi‐copper‐mer catalysts, in which the CuN_4_ rather than Cu atom serves as the basic building blocks. Cu atoms are selected in the present study because the Cu is one of the most demonstrated SAC for CO_2_RR, and has been proven to be able to produce C1, C2, and C3 products.^[^ ^13^, ^15^ ^]^ N atoms around Cu atoms are believed to facilitate the CO_2_ activation or the dissociation of the intermediates. We first synthesize three different types of quasi‐copper‐mer catalysts: quasi‐copper‐monomers, quasi‐copper‐dimers, and quasi‐copper‐trimers hosted in a graphene‐like substrate and perform experiment characterization and then the DFT calculations to examine their atomic structures, evaluate the catalytical performance and understand their underlying physical mechanisms. Our results show that the quasi‐copper‐trimers outperform quasi‐copper‐monomer and quasi‐copper‐dimer such that they give the best activity for CO_2_RR to CO and the best selectivity against the competing hydrogen evolution reaction (HER). By combining the DFT calculation and experimental results, we not only show a promising catalyst for CO_2_RR, but also demonstrate a practically viable route in the design of new catalysts going beyond of single‐atom catalysts.

## 2 Results and Discussion

### 2.1 Structure Characterization

**Figure 1:**
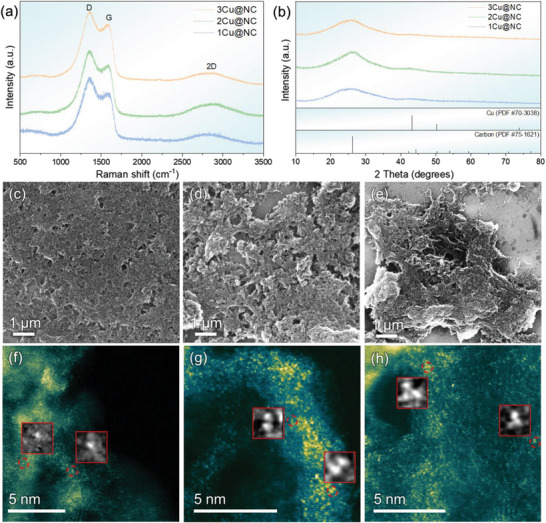
a) Raman spectra of the 1Cu, 2Cu, and 3Cu@NC samples, showing that the carbon is mainly in sp^2^ hybridization with high number of defects. b) XRD image of the 1Cu, 2Cu, and 3Cu@NC samples, showing no crystalline Cu formed. SEM and HAADF‐STEM images of c,f) 1Cu@NC, d,g) 2Cu@NC, and e,h) 3Cu@NC samples, showing the distribution of Cu atoms on carbon matrix. The area in yellow represents bright part, and the area in green represents dark part in the STEM images. The red circles in (f–h) indicate Cu single‐atoms, dimers, and trimers. The insets are the magnified images in the red circles.

In order to synthesize the representative quasi‐copper‐mer samples for the investigation of their CO_2_RR performance, an appropriate substrate is essential in order to provide high population of neighboring anchor sites for Cu atoms, stabilize the Cu atoms, and prevent their aggregation. Therefore, a heavily N‐doped carbon substrate was purposely synthesized through the carbonization of C_3_N_4_. The as‐synthesized C_3_N_4_ exhibits a twisted thin sheet morphology and its X‐ray diffraction (XRD) peaks can be well indexed to the (100) and (002) planes of g─C_3_N_4_,^[^ ^16^ ^]^ which proves the successful synthesis of g─C_3_N_4_ (Figure S1a,b, Supporting Information). Upon the heat treatment, the corresponding XRD peaks of C_3_N_4_ disappeared. Instead, a widened carbon peak at ≈2*θ* of 25° occurred in all three samples. This suggests that C_3_N_4_ had been fully transferred to N‐doped carbon. The CHNS elemental analyzer results also show C_3_N_4_ consists of 34 wt.% C and 61 wt.% N, while the C content in N‐doped carbon is ≈57 wt.% and N content is ≈31 wt.%, which are due to the decomposition of C_3_N_4_ to N‐doped carbon and N_2_ at high temperature. In the corresponding Raman spectrum of the final products (**Figure** **1**a), all three samples exhibited a 2D Raman signal at ≈2670 cm^−1^, which is characteristic of graphitic *sp^2^* hybridized carbon.^[^ ^17^ ^]^ The *I*_D_/*I*_G_ ratios of the three samples are 1.24, 1.28, and 1.30, respectively, suggesting that the carbon contains numerous defects, which would benefit the stabilization of quasi‐copper‐mers. The abundant nitrogen atoms and defects can help anchor and stabilize Cu atoms to assist the formation of quasi‐copper‐mers.

Cu ions were introduced into C_3_N_4_ substrate by the impregnation method, and then converted to atomic Cu species upon the heat treatment of C_3_N_4_ and acid leaching. By tuning the loading of the copper precursors during the impregnation, the spacing between neighboring Cu atoms is controlled. Hence, by raising the loading level, the quasi‐copper‐monomers (1Cu@NC), quasi‐copper‐dimers (2Cu@NC), and quasi‐copper‐trimers (3Cu@NC) can be achieved. In this work, the loading of Cu in the as‐prepared three samples were determined by Inductively Coupled Plasma (ICP) to be 2.43, 3.13, and 3.41 wt.%, respectively. Before the acid leaching, the 3Cu@NC sample shows small Cu peaks and the scanning electron microscopy (SEM) image shows the clear presence of copper particles (Figure S1b,c, Supporting Information), while after acid leaching, XRD results of all three samples only show a carbon peak and no copper metal peak is detected (Figure 1b), suggesting that no crystalline Cu is formed. Copper particles cannot be observed as well in all three samples, as shown in Figure 1c–e. These results indicate that copper metal particles have been leached out and copper atoms are dispersed into the N‐doped carbon matrix without obvious agglomeration. Further investigation of the samples through HAADF‐STEM has revealed the distribution of Cu atoms in the N‐doped carbon substrate. As shown in Figure 1f–h, 1Cu@NC has few Cu atoms, and the atoms are separated from each other. With increasing Cu content, more 2Cu@NCs are formed, and the inter‐spacings between Cu atoms are shorter, and 2Cu sites can be observed on the carbon matrix. Further increasing the Cu content, 3Cu sites can be found in the 3Cu@NC sample, which is indicated by the red circles. This suggests that the number of quasi‐copper‐dimer and quasi‐copper‐trimer sites become increasingly higher when the loading of Cu increases.

**Figure 2:**
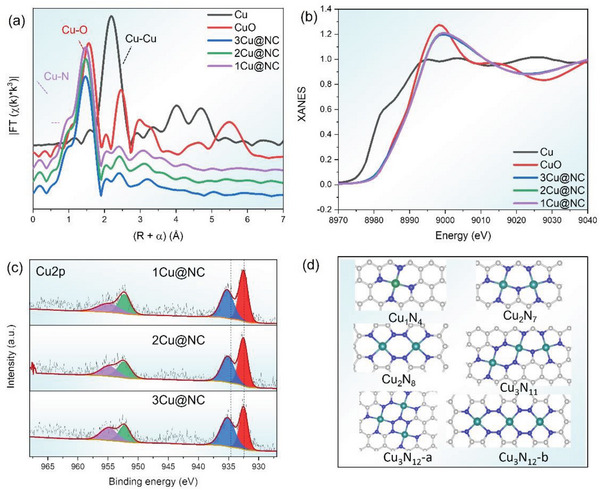
a) EXAFS, and b) XANES spectra of the 1Cu, 2Cu, and 3Cu@NC samples as well as Cu and CuO reference samples, indicating the Cu─N_4_ coordination environment in all three samples. c) XPS spectra of 1Cu, 2Cu, and 3Cu@NC samples, showing that Cu atoms are in the 2+ oxidation state and no Cu metal is found. d) The Cu catalysts studied by DFT calculations. Only those in line with our spectrum results are selected. Gray, blue, and green color spheres correspond to C, N, and Cu atoms, respectively.

The extended X‐ray absorption fine structure (EXAFS) results demonstrate more clearly the local structures of the Cu atoms. More specifically, they reveal that the three samples after acid washing present atomically dispersed Cu, whose main coordination structure can be fitted to Cu─N_4_, as shown in **Figure** **2**a and Figure S2 (Supporting Information). The fitting parameters are shown in Table S1 (Supporting Information). It is seen that even with the increase of Cu loading, each Cu atom still binds with four N atoms and is separated from other Cu atoms. The Cu 2p X‐ray photoelectron spectroscopy (XPS) spectrum shows a characteristic Cu^2+^ peak and its corresponding satellite peak. The X‐ray absorption near‐edge structure (XANES) spectrum also suggests the oxidation state of Cu in the three samples is similar to that of CuO (Figure 2b,c). These results prove again that there is no Cu─Cu direct bond formed and the Cu─N_4_ coordination environment is verified. Further investigation by using high‐angle annular dark‐field scanning transmission electron microscopy (HAADF‐STEM) revealed the distribution of Cu atoms on the N‐doped carbon. As shown in Figure 1f–h, there are few Cu atoms, and there are separated from each other. With increasing Cu content, more Cu atoms appear, and the spacing between Cu atoms become closer. 2Cu sites can well be observed on the carbon matrix. Further increasing the Cu content, 3Cu sites can be found in 3Cu@NC sample, which is indicated by the red circles. The structures of the three samples therefore are in agreement with the quasi‐copper‐mers model and will be used subsequently to evaluate their CO_2_RR performance.

In addition, the preferred formation of quasi‐copper‐mer structures are further supported by our DFT simulations. Figure S3 (Supporting Information) shows the possible geometries of the different copper‐mers models in the 1Cu, 2Cu, and 3Cu@NC samples. The relationship between the neighboring Cu atoms can be classified into two types – direct Cu─Cu interaction and indirect (i.e., quasi‐type) Cu─Cu interaction. As suggested by the EXAFS results discussed above, the coordination structure of Cu single‐atoms is determined to be Cu─N_4_ as shown in Figure 2d, which is also well documented in the available literatures.^[^ ^7a,b^ ^]^ For the quasi‐copper‐dimers, two different geometries are proposed in Figure S3 (Supporting Information). Cu_2_N_8_ structure shows two Cu─N_4_ that are close to each other while Cu_2_N_7_ shows two Cu─N_4_ sharing one N atom. The Cu─Cu distance of 4.05 and 3.65 Å are much longer than the Cu─Cu distance of 2.34 Å, suggesting that these two Cu atoms do not interact directly, which highlights the uniqueness of the quasi‐copper‐dimers. The other Cu dimer of Cu_2_N_6,_ as shown in Figure S3 (Supporting Information), demonstrates a Cu─Cu distance of 2.40 Å, which is close to the experimental 2.34 Å, suggesting that these two neighboring Cu atoms are directly bonded. As for the Cu trimer, seven possible structures were considered with three of them containing indirect Cu─Cu interaction solely (Cu_3_N_11_, Cu_3_N_12_‐a, and Cu_3_N_12_‐b, as shown in Figure S3, Supporting Information), two of them only contain direct Cu─Cu interaction (Cu_3_N_7_ and Cu_3_N_8_), and two of them contain a mixture of direct and indirect Cu─Cu interaction (Cu_3_N_11_ ‐a and Cu_3_N_10_). The formation energy was calculated for the final Cu adsorption to form Cu dimers and trimers. The results are summarized in Figure S4 (Supporting Information), in which the adsorption energy of the Cu to form the quasi‐dimer/trimer is lower than that of the direct dimers/trimers, suggesting that the quasi‐copper‐mer geometries are more stable than those of the direct Cu─Cu interaction geometries. The formation energy calculation thus well supports the EXAFS and XANES results that only quasi‐copper‐mers are detected because they are energetically more favorable. Thus, as shown in Figure 2d–i, only the models with indirect Cu─Cu interaction are selected for further Gibbs free energy calculations.

### 2.2 Catalytical Performance

**Figure 3:**
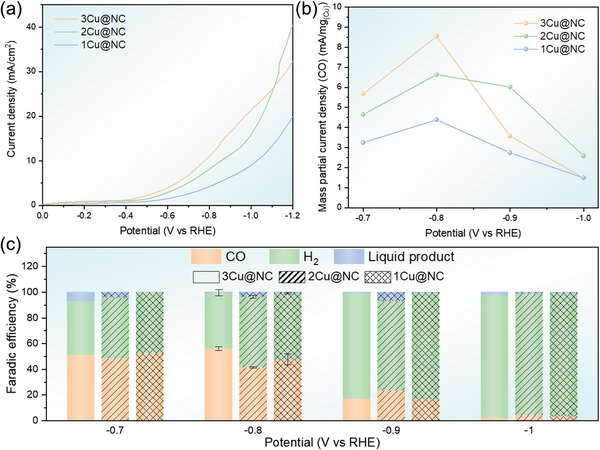
a) Linear sweep voltammetry curves, b) partial current density of CO, and c) faradaic efficiency of CO and H_2_ for the 1Cu, 2Cu, and 3Cu@NC. 3Cu@NC sample shows the highest CO production at −0.8 V, which is higher than both 1Cu and 2Cu@NC samples. The selectivity to CO of 3Cu@NC sample at −0.8 V is also higher than the other two samples. The error bar is shown for potential at −0.8 V.

In the present work, we have successfully synthesized an appropriate substrate with a high population of anchoring sites, together with abundant chemical N doping, to stabilize the Cu quasi‐copper‐mers and prevent their aggregation. We have performed both first‐principles calculations and EXAFS and XANES characterization to support the stability of the quasi‐copper‐mers atomic configurations. Both our theoretical and experimental results provide compelling evidence supporting the stability of the quasi‐copper‐mers. The electrocatalytic activity of the three samples for CO_2_RR was thus performed in an H‐type cell with CO_2_ saturated 0.5 m KHCO_3_ solution as the electrolyte. The gas products were analyzed by gas chromatography analysis. It suggests the gas‐phase products catalyzed by all three samples are only CO and H_2_; both methane and ethylene are not detected in the measurements. The total faradic efficiency for the two products exceeds 90% at all potential. The rest products should be different liquid compounds, which are not focused in this work and termed as “liquid product” to denote all the potential liquid products. Compared to the onset potential of −0.49 V for 1Cu@NC, the 2Cu, and 3Cu@NC exhibit a less cathodic value of −0.43 and −0.38 V, respectively (**Figure** **3**a). In addition, the mass‐specific partial current density has been calculated using the weight ratio of Cu determined by ICP for the three samples before the CO_2_RR measurements, which is similar as the previous literatures.^[^ ^18^ ^]^ 3Cu@NC shows a much higher mass‐specific partial current density of CO as compared to that of the 1Cu@NC sample at the potential of −0.7 to −1 V, and reaches a 100% higher value at −0.8 V. Meanwhile, the mass‐specific partial current density of CO for 3Cu@NC is also 29% higher than that for 2Cu@NC. These measurement results indicate a higher CO_2_ to CO catalytic ability of 3Cu@NC (Figure 3b). Concerning the competition between HER and CO_2_RR, the Faradic efficiency of CO and H_2_ is analyzed and shown in Figure 3c. At the potential of −0.8 V, 3Cu@NC exhibits the lowest HER contribution as compared to the other two samples. Its Faradic efficiency of CO reaches ≈56%, while the values for 1Cu@NC and 2Cu@NC are only 41% and 47%, respectively. When the potential is < −1 V, the majority of the gas product is H_2_ for all three samples, and the faradaic efficiency of CO becomes less than 10%. The electrocatalytic performance results suggest that the selectivity and activity of the 3Cu@NC are better than those of 2Cu@NC and the two samples are both better than 1Cu@NC. Hence, our results suggest that by increasing the number of CuN_4_ units, the catalytic activity, and selectivity of the catalyst can be both enhanced.

To understand the mechanism of such improvement in electrocatalytic performance, DFT calculations have been carried out. Both DFT calculations and experimental techniques have already been extensively applied in studying the CO_2_ reduction to CO on crystals, nanoparticles/clusters, and atomic Cu.^[^ ^19^ ^]^ Previous studies generally agree that *CO_2_ is reduced to ^*^CO via ^*^COOH as the intermediate. Herein, we adopt this reaction pathway in our DFT calculations to explore the catalytical energy landscapes of our quasi‐copper‐mer catalysts.

**Figure 4:**
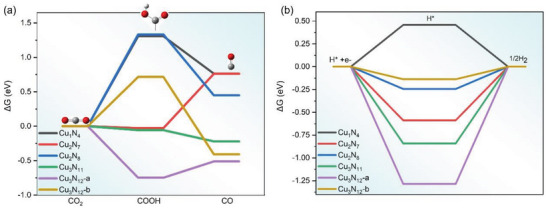
The Gibbs free energy diagram for a) CO_2_RR to CO, and b) HER on quasi‐copper‐monomer (black), quasi‐copper‐dimer (red and blue), and quasi‐copper‐trimer (green, purple, and golden for three different geometries as shown in Figure 2d). Here the quasi‐copper‐dimer (red and blue) shows better activity than quasi‐copper‐monomer (black) and the quasi‐copper‐trimer (green, purple, and golden) group shows the best activity. In terms of selectivity against HER, quasi‐copper‐dimer (red and blue) shows similar performance as quasi‐copper‐monomer (black) which quasi‐copper‐trimer (green, purple, and golden) group shows the best selectivity.

The Gibbs free energy change (Δ*G*) for CO_2_ reduction to CO catalyzed by quasi‐copper‐mers are summarized in **Figure** **4**a and Table S2 (Supporting Information). For 1Cu@NC, the CO_2_ weakly adsorbs on the substrate with *E*_ads_ = −0.13 eV, which is still stronger than the −0.02 to −0.05 eV on Cu surface^[^ ^20^ ^]^ and −0.03 eV on graphene.^[^ ^21^ ^]^ The relatively stronger adsorption energy of CO_2_ would be beneficial for atomic Cu to fix and activate CO_2_, thereby making the reduction reaction more favorable. Here the Δ*G* for ^*^CO_2_ protonation to ^*^COOH is calculated to be 1.31 eV (as shown in Figure 4a, black line and Table S2, Supporting Information), which is in good agreement with the literature value.^[^ ^22^ ^]^ Then, it will undergo an exothermic reaction to ^*^CO with Δ*G* of −0.54 eV. There are two different 2Cu@NCs, namely Cu_2_N_7_ and Cu_2_N_8_, as shown in Figure S3 (Supporting Information). The 2Cu@NC Cu_2_N_7_ performs differently from 1Cu@NC (Figure 4a, red line vs black line) that on top of Cu_2_N_7_, ^*^CO_2_ is reduced to ^*^COOH with an exothermic Δ*G* of −0.03 eV and then ^*^COOH is reduced to ^*^CO with an endothermic Δ*G* of 0.79 eV. The limiting potential (*U*_e_) is 0.79 eV, which is much lower than the *U*_e_ of 1.33 eV for the 1Cu@NC. We expect that 2Cu@NC Cu_2_N_7_ is more active than 1Cu@NC for CO_2_RR to CO. The 2Cu@NC Cu_2_N_8_ performs similarly with 1Cu@NC with Δ*G* of 1.33 and −0.88 eV, when catalyzing CO_2_RR to CO. It is difficult to determine the quantity of these two 2Cu@NCs, considering that Cu_2_N_7_ is more active than 1Cu@NC and Cu_2_N_8_ performs similarly to 1Cu@NC. We, therefore, conclude that the 2Cu@NC presents a higher activity than that of 1Cu@NC due to the combined effect of Cu_2_N_7_ and Cu_2_N_8_. The catalytical performance for 3Cu@NCs is remarkable. As shown in Figure 3a and Table S2 (Supporting Information), the 3Cu@NC Cu_3_N_11_ demonstrates an exothermic Δ*G* of −0.06 eV when catalyzing ^*^CO_2_ to ^*^CO, and then to ^*^COOH on Cu_3_N_11_ by further releasing an energy of 0.16 eV to obtain the final product of ^*^CO. Both reaction steps are exothermic, which indicate that the CO_2_RR to CO on Cu_3_N_11_ is thermodynamically favorable. Cu_3_N_12_‐a demonstrates an exothermic Δ*G* as low as −0.75 eV for ^*^CO_2_ to ^*^COOH and then needs 0.24 eV to the final product of ^*^CO. The Δ*G* for CO2RR to CO on Cu_3_N_12_‐b via ^*^COOH is 0.72 and −1.22 eV. The limiting potential is 0.72 eV, which is lower than the 0.79 and 1.33 eV for the two 2Cu@NCs as well as the 1.31 eV for 1Cu@NC.

Our DFT calculations clearly show that the quasi‐copper‐trimer outperforms the quasi‐copper‐monomer and quasi‐copper‐dimer when catalyzing CO_2_RR to CO. It is of interest to note that the 3rd Cu atom is of significant importance in enhancing the activity. The C_2_N_7_ (Figure 2d) gives a Ue of 0.79 eV while the 3rd Cu (as in C_3_N_11_,) makes the whole process thermodynamically favorable. Similarly, the C_2_N_8_ gives a Ue of 1.33 eV while the 3rd Cu (as in C_3_N_12_‐b) effectively lowers the Ue to 0.72 eV. To further reveal the underlying mechanism responsible for the changes, we have performed *p*‐band center calculations on the active sites of all the catalysts (Figure S5, Supporting Information). Interestingly, a volcano curve is revealed, which can explain the activity trend of all these Cu mers catalysts based on the understanding that the one with its *p*‐band center closer to the optimum value gives a better activity. Importantly, the role of the 3rd Cu atom is also revealed: The 3rd Cu atom is able to move the active site's *p*‐band center toward the optimum value, thus giving rise a better activity.

The competition between CO_2_RR and HER is another major concern when designing highly efficient catalysts for CO_2_RR, as HER can lower the CO_2_RR selectivity and efficiency.^[^ ^23^ ^]^ Therefore, a good catalyst should exhibit appropriate electronic properties which not only favor the CO_2_RR but also dramatically suppress the competitive HER. Herein, we have compared the selectivity performance of these quasi‐copper‐mer catalysts against HER. As shown in Figure 4b and Table S3 (Supporting Information), the 1Cu@NC demonstrates a positive Δ*G* of 0.46 eV, while 2Cu@NCs Cu_2_N_7_ and Cu_2_N_8_ demonstrate a Δ*G* of −0.59 and −0.24 eV, respectively. On the one hand, it is hard to conclude whether the 2Cu@NC is more active than 1Cu@NC for HER as it is dependent on the geometry of 2Cu@NC (Cu_2_N_7_ or Cu_2_N_8_). On the other hand, 3Cu@NCs Cu_3_N_11_ and Cu_3_N_12_‐a exhibit a high Δ*G* of −0.84 and −1.29 eV, respectively. Such low Δ*G* values indicate that they are not active in HER reaction, thus be beneficial for CO_2_RR. Hence, the 3Cu@NCs Cu_3_N_11_ and Cu_3_N_12_‐a are expected to possess a good selectivity against HER. However, the 3Cu@NC Cu_3_N_12_‐b demonstrates a Δ*G* of −0.14 eV, which is the closest to the zero line, indicating that it is active in HER. In summary, 3Cu@NCs Cu_3_N_11_ and Cu_3_N_12_‐a demonstrate their robustness against HER because they exhibit a high Δ*G* (−0.84 and −1.29 eV, respectively). And the small Δ*G* (−0.14 eV) suggests that 3Cu@NC Cu_3_N_12_‐b is active in HER. Since in the real experiments, the quasi‐copper‐trimer sample can be a mixture of all these different geometries, we can still expect that the 3Cu group demonstrates a better selectivity than 1Cu@NC and 2Cu@NC.

Both the DFT calculations and the experimental performance results show that 3Cu@NCs have both higher activity and selectivity to CO than 2Cu@NCs and 1Cu@NC. This demonstrates that by increasing the number of CuN_4_ building blocks, the CO_2_RR performance can be enhanced. Hence, quasi‐metal‐mers present a great potential to improve the current catalytical performance. In the present study, we have studied the quasi‐copper‐mers up to three CuN_4_ units. Clearly, the properties of the sample with more CuN_4_ units are worth being investigated in the future. In addition, the atomic arrangement of quasi‐copper‐mers also plays an important role in the electrocatalytic CO_2_RR performance. Establishing a precise control method to specifically synthesize one type of quasi‐metal‐mers will be another interesting topic worth further investigation.

## 3 Conclusion

Going beyond the recently established SACs, where single atomic entity functions, we proposed a new class of electrocatalysts: quasi‐copper‐mers embedded in an N‐doped graphene‐like substrate, and investigated their atomic structures and electrocatalytic performance for CO_2_RR to CO by both experimental characterizations and DFT calculations. First, we synthesized the quasi‐copper‐mer catalysts and demonstrated that the effectiveness of the indirect Cu─Cu quasi‐copper‐mers in electrocatalysis, including both quasi‐copper‐dimers and quasi‐copper‐trimers. We then performed DFT calculations on various potential structures with both direct and indirect Cu─Cu interactions, and shown that the indirect Cu─Cu quasi‐copper‐mer structures are thermodynamically more favorable, thus supporting our experimental observations. Our performance characterization results showed that the quasi‐copper‐trimer outperforms quasi‐copper‐monomer and quasi‐copper‐dimer when catalyzing CO_2_ to CO, which is consistent with the energy landscape of CO_2_RR from our DFT calculations. More interestingly, quasi‐copper‐trimers exhibit a 2‐times higher partial current density of CO than that of quasi‐copper‐monomer. Meanwhile, the ratio of Faradic efficiency of CO to H_2_ for quasi‐copper‐trimer sample is also the highest among all three samples, at −0.8 V. Hence, we conclude that Cu quasi‐trimers outperform both quais‐copper‐monomer and quasi‐copper‐dimer when electrocatalyzing CO_2_RR to CO. The *p*‐band center calculations explain the activity trend based on the understanding that the one with its p‐band center closer to the optimum value gives a better activity. It also highlights the important role of the 3rd Cu atom that can move the *p*‐band center of the active site toward the optimum value, thus giving rise to a better activity. The present work presents a novel route in the design of new electrocatalysts for CO_2_RR to CO with high activity, selectivity, and stability.

## 4 Experimental Section

### Density Functional Theory (DFT)

All the calculations were performed based on DFT with the Perdew–Burke–Ernzerhof functional under the generalized gradient approximation^[^ ^24^ ^]^ for the exchange‐correlation interaction, as implemented in the Vienna Ab initio Simulation Package (VASP).^[^ ^25^ ^]^ A cutoff kinetic energy of 500 eV was applied to expand the electronic wave functions and the projector augmented‐wave method was adopted to describe the electron‐core interaction. A Gamma centered 3 × 3 × 1 k‐mesh was used for the structural optimization. The Gibbs free energy change (Δ*G*) at each electrochemical step involving a proton–electron transfer was computed based on computational hydrogen electrode (CHE) model, in which the free energy of (H^+^ + 𝑒^−^) equals to $\frac{1}{2}H_{2}\left(g\right)$ for standard hydrogen electrode (SHE).^[^ ^26^ ^]^ So the Δ*G* of each reaction step is defined as:

(1) $$\Delta G=\Delta E_{adsorp}-\Delta E_{ZPE}-T\Delta S$$

where Δ*E*_adsorp_ is the adsorption energy differences between the product adsorbate and reactant adsorbate, Δ*E*_ZPE_ is the difference in zero‐point energy, *T* is the temperature (300 K) and Δ*S* is the entropy difference between the adsorbed adsorbate and non‐adsorbed gas‐phase adsorbate. Δ*G*describes the energy needed or released for a reaction to occur. A positive Δ*G* suggests an endothermic reaction, while a negative Δ*G* suggests an exothermic reaction. It is understood that the higher the Δ*G* is, the more energy is needed for a reaction to take place. To better mimic the real experiments, solvation effects have been included by using an implicit model.^[^ ^27^ ^]^

### Preparation of g─C_3_N_4_

A covered ceramic crucible filled with urea was heated in a muffle furnace at 525 °C for 4 h at the heating rate of 5 °C min^−1^. The yellowish g─C_3_N_4_ powder was then obtained and grinded.

### Preparation of 1Cu, 2Cu, and 3Cu@N‐Carbon

g─C_3_N_4_ (4.4 g) and 3.6 g of Pluronic F127 were added into 360 mL of deionized (DI) water. The mixture was then sonicated for 1.5 h and stirred for 1.5 h to achieve uniform dispersion. Afterward, 20 mL of 0.2 m, 0.5 m, and 1 m CuCl_2_ aqueous solution were added drop‐by‐drop into the above mixture respectively and stirred overnight. The dispersion was collected and washed using DI water three times by centrifugation at 8000 rpm for 5 min each. After drying, the powder thus‐obtained was calcinated at 550 °C for 2 h and 800 °C for 1 h at the heating rate of 3 °C min^−1^ in Argon atmosphere. Afterward, 10 mg of the obtained product was immersed in 20 mL of 2 m HCl solution for 6 h with stirring to leach out the copper particles and clusters. 1Cu, 2Cu, and 3Cu@N‐carbon were then obtained after filtering, washing, and drying.

### Characterization

The morphology and structure of the prepared samples were characterized by using scanning electron microscopy (Zeiss Supra 40) and scanning transmission electron microscopy (JEOL ARM200F). X‐ray diffraction analysis studies were operated using Bruker D8 diffractor at 40 kV and 40 mA with Cu K radiation (0.15 406 nm). Raman spectroscopy was performed using HORIBA LabRAM HR Evolution Raman microscopes with an Argon laser (*λ* = 514 nm, National Laser Model 800AL) as the excitation line. The X‐ray photoelectron spectroscopy tests were conducted using Kratos Analytical Axis Ultra DLD UHV. The results were callibrated by alinging the carbon 1s peak to 284.6 eV. The elemental composition were determined by using the ThermoFisher Scientific FlashSmart CHNS Elemental Analyzer and ICP (Perkin Elmer Avio 500). The X‐ray absorption spectra (XAS) including X‐ray absorption near‐edge structure and extended X‐ray absorption fine structure of the samples at Cu K‐edge were collected at the XAFCA beam line of the Singapore Synchrotron Light Source (SSLS), where a pair of Si (111) crystals was used in the monochromator. The XAS data were recorded in a transmission mode. Cu foil, Cu, and CuO were used as references. The storage ring was working at the energy of 700 M eV with an average electron current of 200 mA.

### Electrochemical Measurements

The electrochemical studies were performed in an H‐type cell using the electrochemical workstation (CHI 760E). Pt mesh and Ag/AgCl electrode (3.5 m KCl) were used as the counter electrode and reference electrode, respectively. 4.5 mg of catalysts were suspended in 440 µL ethanol solution with 10 µL Nafion added. Then, 100 µL of catalyst ink was drop cast onto a carbon paper (1 × 1 cm^2^). The mass loading of the catalyst on working electrodes was 1 mg cm^−2^. A CO_2_‐saturated 0.5 m KHCO_3_ (pH ≈7.22) was used as the electrolyte with continuous CO_2_ supply at the flow rate of 23 mL min^−1^. The volume of electrolyte was 30 mL for both anode and cathode chambers in the H‐type cell. All potentials measured were calibrated to the reversible hydrogen electrode (RHE) reference scale using *E*_RHE_ = *E*_Ag/AgCl_ + 0.0591 × pH + 0.2046. Gas products were analyzed by using the online gas chromatograph (Shimadzu, 2014C). H_2_ was detected by a thermal conductivity detector, and CO was detected by a flame ionization detector. The Faradaic efficiency was calculated based on the equation below,

(2) $$FE=\frac{nvpVF}{IRT}\times 100\%$$

where *n* is the electron transfer number, *v* is the volume concentration of examined gas in the outlet gas, p is the atmospheric pressure (1.013 × 10^5^ Pa), *V* is the gas flow rate, *F* is the Faradic constant, *I* is the steady‐state total current density, *R* is the ideal gas constant, *T* is the room temperature (298.15 K). The test at potential of −0.8 V has been repeated for three times for calculating the error bar.

## Conflict of Interest

The authors declare no conflict of interest.

## Supporting information

Supporting Information

## Data Availability

The data that support the findings of this study are available from the corresponding author upon reasonable request.
