# Supplementary material for: Quasi‐Copper‐Mers Enable High‐Performance Catalysis for CO2 Reduction
Source: Adv Sci (Weinh). 2023 Aug 8;10(29):2303297. doi: 10.1002/advs.202303297 (PMC10582455; doi:10.1002/advs.202303297)
Supplement: Supplementary file 1 — Supporting Information [file ADVS-10-2303297-s001.pdf]

ADVANCED SCIENCE
Open Access

Supporting Information

for *Adv. Sci.*, DOI 10.1002/advs.202303297

Quasi-Copper-Mers Enable High-Performance Catalysis for CO_2 Reduction

Jing Yang, Ximeng Liu, Zhao Li, Shibo Xi, Jianguo Sun, Hao Yuan, Weihao Liu, Tuo Wang, Yulin Gao, Haimei Wang, Junjie Wang, Jun Song Chen, Rui Wu, Yong-Wei Zhang and John Wang**

Supporting Information

Quasi-copper-mers Enable High-Performance Catalysis for CO_2 Reduction

Jing Yang[1‡], Ximeng Liu[2‡], Zhao Li[3], Shibo Xi[4], Jianguo Sun[2], Hao Yuan[1], Weihao Liu[2], Tuo Wang[2], Yulin Gao[2], Haimei Wang[2], Junjie Wang[3], Jun Song Chen[3], Rui Wu[3], Yong-Wei Zhang[1], John Wang[2*]*

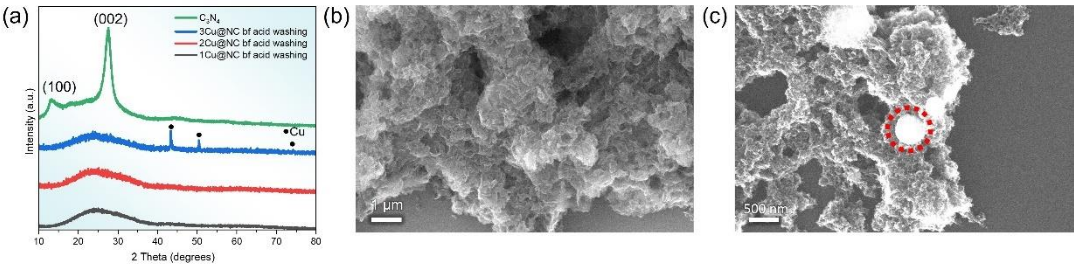

Figure S1. (a) XRD images of C_3N_4, 1Cu@NC, 2Cu@NC, and 3Cu@NC before acid leaching. SEM images of (b) C_3N_4 and (c) 3Cu@NC before acid leaching. The red circle in (c) indicates an example of copper particles.

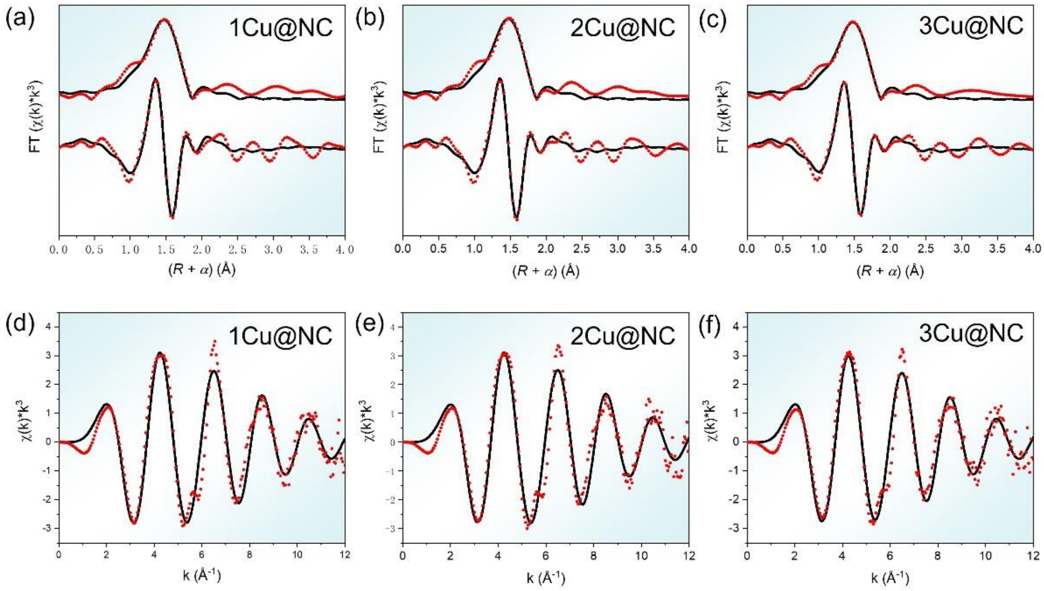

Figure S2. Cu K-edge EXAFS (points) and fitting curve (line) for (a) 1Cu@NC , (b) 2Cu@NC, and (c) 3Cu@NC, in *R*-space (FT magnitude and imaginary component). The data are k^3 weighted and not phase-corrected. Cu K-edge EXAFS (points) and fitting curve (line) for (d) 1Cu@NC , (e) 2Cu@NC, and (f) 3Cu@NC, in k^3 weighted *K*-space.

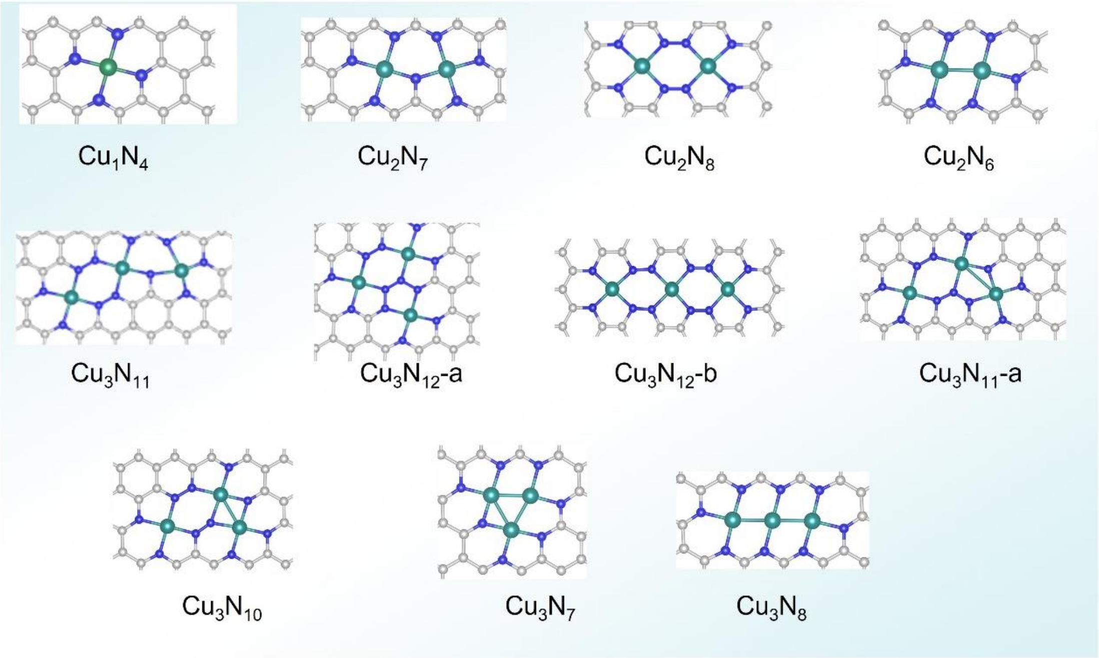

Figure S3. Summary of possible quasi-copper-monomer, -dimers and -trimers. Gray, blue and green color spheres correspond to C, N and Cu atoms, respectively.

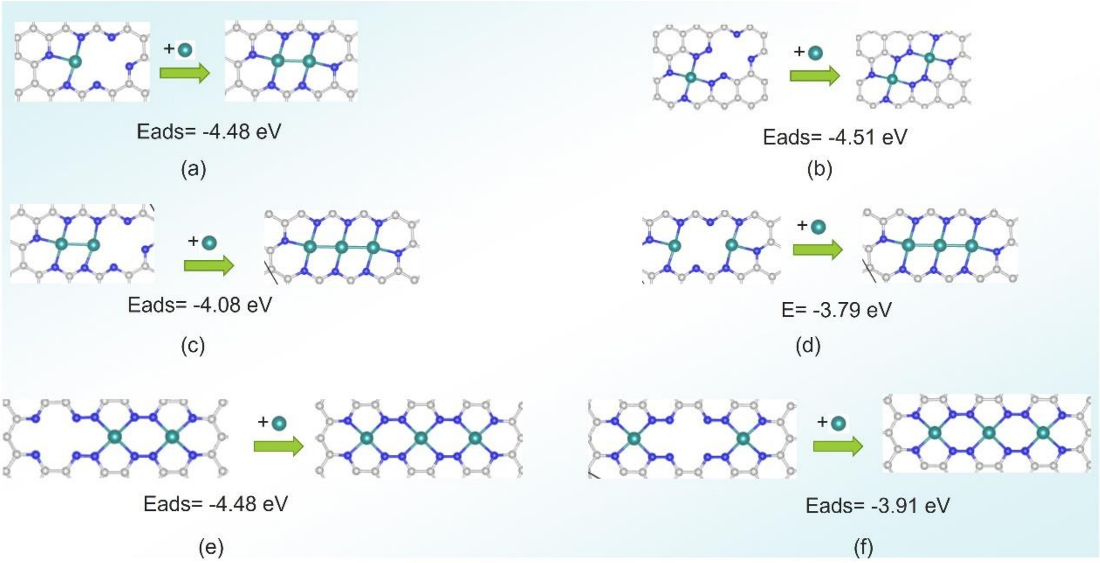

Figure S4. The schematic process to form the direct copper-mers and indirect quasi-copper-mers. The adsorption energy for each step is also listed. Gray, blue and green color spheres correspond to C, N and Cu atoms, respectively.

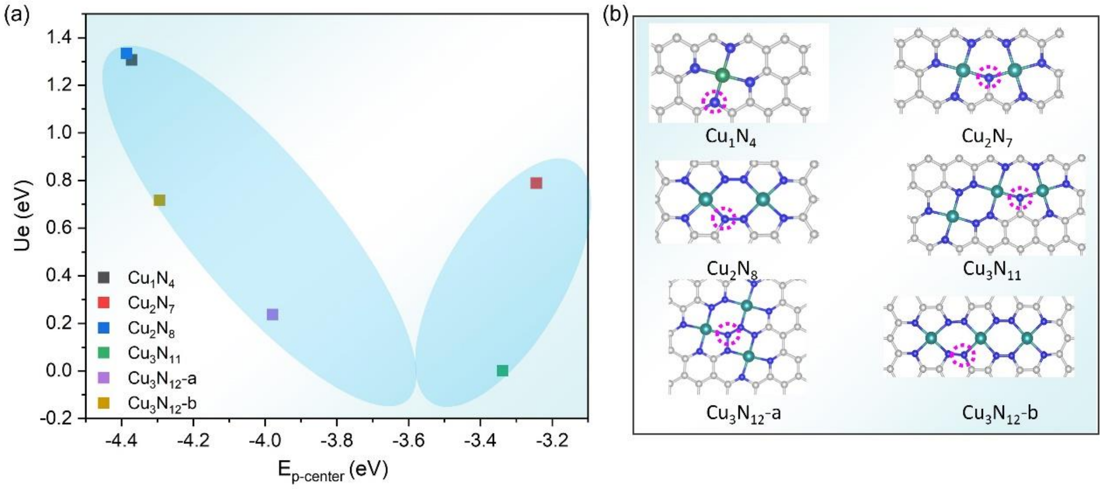

Figure S5. (a) The limiting potential of CO_2RR on all the quasi-copper-mers as a function of the *p*-band center of the active site, as shown in (b).

Table S1. Fitting parameters[a] for Cu K-edge EXAFS for 1Cu@NC , 2Cu@NC, and 3Cu@NC.

Sample	Path	N	R (Å)	σ^2 (Å^2)	ΔE
1Cu@NC	Cu-N	4.5±0.4	1.94±0.01	0.009±0.002	-0.74±0.97
2Cu@NC	Cu-N	4.4±0.4	1.94±0.01	0.008±0.001	-0.61±0.97
3Cu@NC	Cu-N	4.2±0.3	1.95±0.01	0.009±0.001	-0.10±0.75

[a] S_0^2 was fixed as 1. ΔE was refined as a global fitting parameter. Data range: $2.5 \leq k \leq 12$, $1 \leq R \leq 2.5$. The number of variables is 4, out of a total of 8.9 independent points. R factor for 1Cu fit is 1.2 %, for 2Cu is 1.3%, for 3Cu is 0.8%.

Table S2. ΔG (in eV) for each reaction step for CO_2RR to CO on quasi-copper-mers.

Cu catalysts	$*CO_2$+H = *COOH	*COOH+H= *CO + H_2O
Cu_1N_4	1.31	-0.54
Cu_2N_7	-0.03	0.79
Cu_2N_8	1.33	-0.88
Cu_3N_{11}	-0.06	-0.16
Cu_3N_{12}-a	-0.75	0.24
Cu_3N_{12}-b	0.72	-1.12

Table S3. ΔG (in eV) for HER on quasi-copper-mers.

Cu catalysts	ΔG
Cu_1N_4	0.46
Cu_2N_7	-0.59
Cu_2N_8	-0.24
Cu_3N_{11}	-0.84
Cu_3N_{12}-a	-1.29
Cu_3N_{12}-b	-0.14

Table S4. The adsorption energy (E_{adsorp}) of COOH and CO (in eV) on Cu SAC and quasi-copper-mers @Graphene

Cu catalysts	E_{adsorp}(COOH)	E_{adsorp}(CO)
Cu_1N_4	-1.29	-0.15
Cu_2N_7	-2.94	-0.53
Cu_2N_8	-2.14	-0.95
Cu_3N_{11}	-3.28	-1.55
Cu_3N_{12}-a	-3.54	-1.60
Cu_3N_{12}-b	-2.58	-1.90
